# Augmented Reality Visualization and Navigation Operationalized in Biopsy of Indeterminate Splenic Mass

**DOI:** 10.1002/ccr3.71850

**Published:** 2026-01-18

**Authors:** Alexander S. Misono

**Affiliations:** ^1^ Hoag Memorial Hospital Presbyterian Department of Radiology, Division of Interventional Radiology Newport Beach California USA

**Keywords:** 3‐dimensional, augmented reality, computed tomography, electromagnetic, head up display, head‐mounted display

## Abstract

This case report describes the successful use of an augmented reality (AR) guidance system (XR90, MediView XR Inc., Cleveland Ohio) to assist in the percutaneous biopsy of a splenic mass performed in a minor procedure room. Traditional two‐dimensional (2D) imaging modalities such as ultrasound and computed tomography (CT) present limitations in depth perception, spatial orientation, and procedural ergonomics, particularly in anatomically complex and mobile regions like the spleen. The AR system integrates real‐time ultrasound, electromagnetic (EM) tracking, and pre‐procedural CT data to generate a 3D holographic overlay within a heads‐up display (HUD), enhancing operator spatial awareness and needle control. A 69‐year‐old male with a 6 cm splenic mass underwent biopsy using the AR platform alongside ultrasound. The system enabled accurate needle placement, reduced cognitive load, and provided continuous visual confirmation during the procedure. All biopsy samples were successfully obtained on the first pass without complications, and pathology confirmed high‐grade B‐cell lymphoma. This case highlights the feasibility and clinical utility of deploying AR‐guided interventions in outpatient procedure rooms, supporting broader adoption of AR technologies in settings beyond the operating room or angiography suite.

## Key Clinical Message

Augmented reality (AR) provided enhanced 3D visualization and precision guidance during biopsy of a splenic mass, helping to overcome limitations of traditional 2D imaging. By improving spatial awareness in complex anatomy, AR reduced cognitive load and supported safe, efficient needle placement in a minimally invasive procedural setting.

## Graphical abstract

First‐person intraprocedural view of XR90 providing a live holographic 3D rendering of a spleen, splenic mass, and nearby lung parenchyma is used to drive procedural planning, allow for biopsy instrument tracking, define optimal trajectory, and subsequently visualize parenchymal tract closure with SinglePass biopsy tract electrocautery.

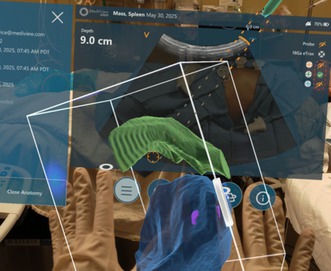

## 1 Introduction

### 1.1 Background

Clinicians performing percutaneous soft tissue biopsies rely on traditional medical imaging tools, predominantly 2D displays from ultrasound (US) and computed tomography (CT) to navigate through complex three‐dimensional (3D) anatomical spaces. This reliance on two‐dimensional views during three‐dimensional procedures often leads to increased cognitive and physical loads on proceduralists, potentially impacting precision and workflow efficiency [1]. Augmented reality (AR) technologies have emerged as promising tools to address these challenges by enhancing spatial orientation and visualization during minimally invasive needle‐based procedures [2, 3]. Recent cadaver and porcine preclinical studies using an AR system in percutaneous needle‐based procedures have shown that AR systems are as accurate and safe as procedures with traditional 2D image guidance, while significantly reducing needle repositions [2, 3, 4]. AR systems have also shown comparable accuracy, increased visualization, decreased radiation dose, and decreased procedure times in the neuro and orthopedic space [5, 6, 7, 8, 9]. Additional demonstrated benefits of AR system utilization are improved ergonomics, reduced operative time, and decreased blood loss [5]. Nearly all existing AR systems are designed to work within traditional operating rooms, interventional angiography suites, and/or hybrid operating room environments. The inherent complexity of intervention in these settings is an excellent match for the technological advances provided by AR systems. Furthermore, operating rooms and angiography suites typically feature tables built without metal components, a feature that is required for radiofrequency energy emission and interpretation that drives AR registration.

While AR systems have been proven to work in such settings for the purposes of supporting complex procedures, there is also a desire to perform AR procedures in minor procedure room settings, defined as designated spaces for performing outpatient procedures. Procedures in these settings are typically performed in short duration and may only require local anesthesia or moderate sedation. These rooms allow for faster procedural and patient turnover as compared with operating room or angiography suites. In interventional radiology, common procedures include paracentesis, thoracentesis, and biopsy procedures. In these settings, many ultrasound‐guided procedures are performed including those with complex or less familiar anatomy. The ability to bring AR into minor procedure room settings will allow for further democratization of this technology.

We present a case in which we performed a splenic biopsy utilizing an AR System as an adjunct to ultrasound (US)‐guided needle biopsy. Splenic biopsies are uncommon and present risks to the patient and challenges to the physician [3]. The spleen's vascularity introduces an increased risk for life‐threatening bleeding and hemorrhage following the procedure. Additionally, pneumothorax, pleural effusion, and colonic injury are known complications [10, 11]. The anatomical position of the spleen, situated in the upper abdominal cavity and partially obscured by the rib cage, complicates access and visualization, making the procedure technically demanding for the physician. The AR system supported precision‐guided needle biopsy along with 3D visualization to overcome the restrictive nature of the organ's location.

### 1.2 Technology Overview

Recent technological advances such as lightweight head‐mounted displays, along with tracked instruments (optical or EM) have led to an increased application of AR devices for clinical interventions across multiple specialties within the surgical and interventional space. AR devices have been successfully used for orthopedic, neurologic, surgical, urologic, and interventional radiology. Table 1 demonstrates a brief overview of recent literature published demonstrating the diverse implications for AR for intraprocedural guidance.

**TABLE 1:** Representative applications of augmented reality in spine surgery, neurosurgery, orthopedics, and interventional radiology.

Clinical specialty	Clinical procedure	Augmented reality (AR) application	Key findings	Citation
Spine surgery	Percutaneous vertebroplasty	AR/AI guided trocar insertion with motion compensation	Procedure was found to be accurate, safe, and lower patient radiation exposure as compared to SOC	Auloge et al. [6]
Spine surgery	Thoracolumbar pedicle screw instrumentation	Intraoperative 3D imaging and registration with AR‐Head Mounted Display (HMD) computer navigation for screw placement	Clinically accurate insertion of pedicle screws	Liu et al. [12]
Neurosurgery	Neurosurgical navigation for brain tumors located on the brain surface	AR based neurosurgical navigation with 3D MRI images viewed through smart glasses	Clear visualization of the surgical field with a clinically acceptable target registration error	Maruyama et al. [9]
Neurosurgery	Craniotomy for intracranial tumors and vascular lesions	AR neuro navigation with 3D MRI/CT reconstructions overlaying the surgical field via microscope‐ or HMD‐based visualization	AR facilitated visualization of cortex and critical structures. Preserved neurological function with no AR related complications in gross and near total resection	Gómez et al. [13]
Orthopedics	Minimally invasive Scapula fracture surgery	Pre‐operative virtual simulation combined with intra‐operative navigation assisted fixation	Decreased procedure times and less blood loss as compared to SOC	Guo et al. [5]
Orthopedics	Acetabular cup placement during total hip arthorplasty	AR system produced superimposed image in the surgical field via smarthphone	The AR system provided more accurate information as compared to the conventional method	Ogawa et al. [14]
Interventional Radiology	Percutaneous tumor ablation of soft tissue tumors	AR‐HMD intraprocedural holographic guidance and navigation	Intraprocedural 3D guidance agreed with the standard imaging guidance	Gadodia et al. [2]
Interventional Radiology	Percutaneous Ct‐guided bone biopsy	AR‐HMD intraprocedural lesion targeting and needle trajectory planning	CT passes and radiation does were significantly lower for the AR‐HMD group as compared to SOC	Albano et al. [15]

The benefits for AR have been well charactered for specialties with fixed locations such as neurologic or orthopedic applications. However utilizing AR for percutaneous soft tissue with which are often affected by soft tissue deformation and respiratory motion require complex solutions. One such solution is the XR90 (MediView, Cleveland, OH) system which integrates live ultrasound imaging with multimodal image fusion into a head mounted display along with tracked tools for soft tisse applications.

This platform integrates several cutting‐edge technologies to deliver its unique capabilities. The system utilizes HoloLens 2 (Microsoft, Redmond, WA) as its foundational AR Head‐Mounted Display (HMD), providing an immersive visual interface for clinicians. For real‐time tracking and precise navigation, the AR system incorporates the Aurora Electromagnetic Tracking system (Northern Digital Inc., Waterloo, Canada), which works in conjunction with electromagnetically (EM) sensor‐equipped instruments, including eTRAX (CIVCO Medical Solutions, Coralville, IA) needles and ultrasound probe bracket. Integration with commercially available ultrasound systems, such as the Vivid IQ (GE Healthcare, Chicago, IL), allows for the streaming of live ultrasound imaging to a HUD that is displayed in space. Through a registration process, 3D CT‐based models of segmented patients' anatomy are projected and registered in the procedural space.

## 2 Case Presentation

### 2.1 Patient Profile

A 69‐year‐old male presented with left‐sided abdominal pain and was found to have a 6 cm heterogeneous mass within the spleen. The patient had no significant past medical history and no history of malignancy. Other than the splenic mass, the only other finding on physical examination and imaging was nonspecific, unenlarged retroperitoneal lymph nodes. Medical oncology consulted and a splenic mass biopsy was requested to be performed in interventional radiology.

**FIGURE 1:**
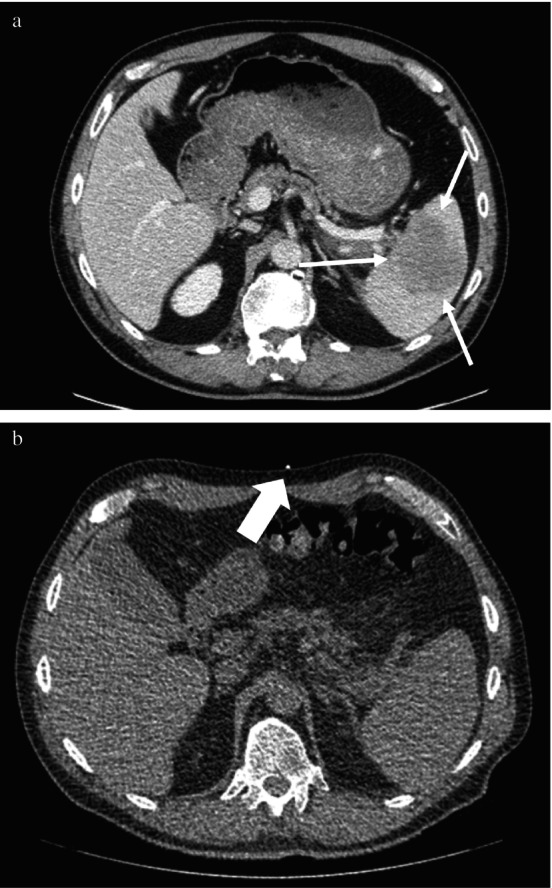
(a) Hypovascular, hypoattenuating splenic mass (white arrows) is seen within the splenic parenchyma. The spleen is not enlarged and is located posteriorly within the upper abdominal cavity. (b) Noncontrast CT imaging reveals that the splenic mass is isoattenuating with splenic parenchyma and cannot easily be visualized. Large white arrow indicates one of three MediView XR90 skin markers required for registration.

Interventional radiology review of the case revealed a normal sized spleen tucked posteriorly within the upper abdominal cavity. There was a 6 cm hypoattenuating, hypoenhancing mass within the splenic parenchyma (Figure 1a). On non‐contrast imaging, however, the splenic mass was poorly defined (Figure 1b).

While a CT‐guided biopsy could be performed using landmarks, given the splenic location, vascularity, and depth, precise needle trajectory planning and real‐time visualization were deemed important to minimize procedural risks and maximize technical success. The patient provided informed consent for the procedure and the use of the AR system.

### 2.2 Procedure Overview

This procedure was completed by an Interventional Radiologist with less than 1 year of AR experience. AR system specific benchtop training was completed prior to clinical procedures. The proceduralist participated in only 2 clinical cases utilizing the AR system prior to this highlighted case, highlighting ease of use. Training was performed rapidly using a phantom model provided by the vendor and consisted of an approximately 20‐min walk‐through of a pre‐existing example case.

Pre‐procedural imaging for the procedure was conducted using a conventional CT scanner. Three radio‐opaque markers were placed on the patient in the left upper quadrant (Figure 1b). Axial CT images with 1.25 mm thick reconstructions were obtained and transferred to the Picture Archiving and Communications System. Following the scan, the radio‐opaque markers were replaced with permanent surgical skin markings, after which the patient was transported to the minor procedure room.

On a separate workstation, the axial CT images were downloaded and imported into a 3D medical imaging post‐processing software (Tera Recon, Concert AI, Durham, NC). Within this workflow, the three radio‐opaque markers, the lung parenchyma of the left lower lobe, the spleen, and the splenic mass were individually contoured. Each of these segmented 3D datasets was then exported in an OBJ file format and subsequently uploaded to the AR system. With the pre‐procedural data prepared, the intervention began.

In the minor procedure room, the patient was placed onto a SurgiGraphic 6000 image‐guided surgical table (Steris, Mentor, OH), followed by sterile prep and draping of the left upper quadrant. Sterile registration markers were then placed on the skin markings. The operator donned the headset and AR registration was initiated and completed using verbal registration commands to the system.

**FIGURE 2:**
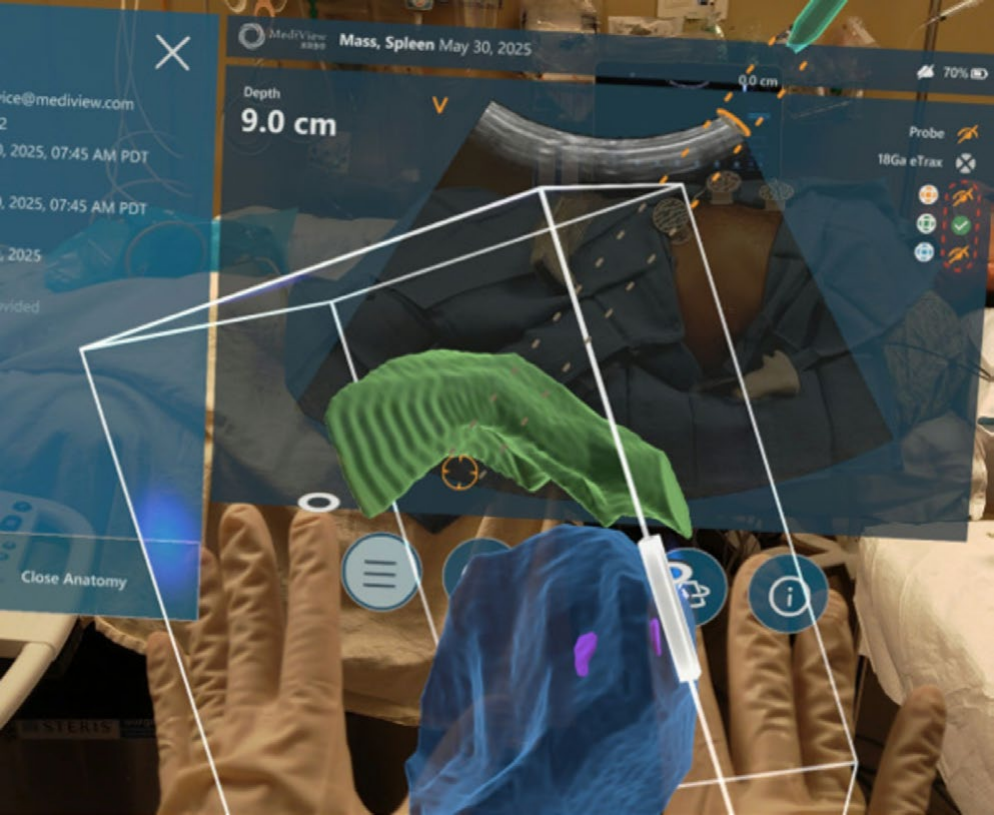
Treatment planning is pursued external to the patient. AR visualization of structures of interest in this case, spleen (blue), splenic mass (purple—partially visualized), and the lower lung (green) allows for dynamic manipulation of structures and review of potential procedural trajectories and plans.

**FIGURE 3:**
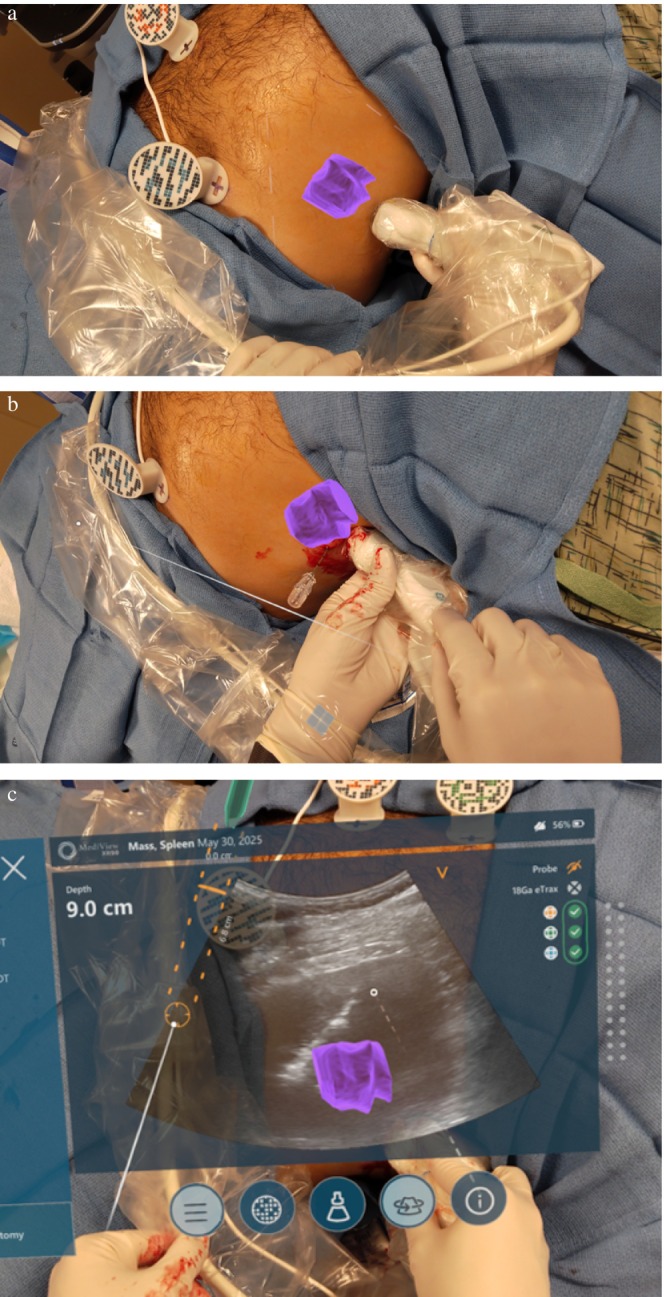
(a) Splenic mass (purple) is visualized in AR onto and within the patient's anatomy. (b) 17‐gauge biopsy guide needle is advanced into the mass under sonographic and AR guidance. (c) Real‐time sonographic guidance of a percutaneous biopsy pass closure with SinglePass Kronos electrocautery is now visualized via a “heads up display” while retaining AR guidance of the splenic mass within the patient torso.

Prior to biopsy initiation, a 3D rendering of the spleen, splenic mass, and lungs was visualized holographically for operator familiarization and treatment planning in open space (Figure 2). Subsequently, this 3D rendering and tracked tools were then registered to the electromagnetic (EM) coordinate space overlaying the 3D structures' patient's anatomy within the EM field to guide biopsy. Upon identifying the optimal trajectory, extraneous structures were removed with verbal command to localize the mass (Figure 3a). A 17‐gauge biopsy guide needle was then advanced into the splenic mass using combined AR and ultrasound guidance (Figure 3b). Core needle biopsies were subsequently performed using sonographic guidance via the head up display feature, allowing for continuous dual confirmation of needle positioning with both sonography and AR throughout the remainder of the procedure.

Three core needle biopsy passes were performed and three core needle specimens were successfully obtained using an 18‐gauge biopsy device (Bard Max‐Core, Becton Dickinson, Franklin Lakes, NJ). The needle track was closed with an electrocautery device (Single Pass Kronos, Single Pass, Lake Forest, CA), with clear delineation of the cauterization tract on final imaging (Figure 3C). Total procedural duration was 15 min.

The patient was observed for 4 h under bedrest restrictions and then discharged home without complications. Biopsy specimens were diagnostic. Final pathology confirmed adequate and diagnostic specimens, revealing high‐grade B‐cell lymphoma, leading to the initiation of treatment. No intra‐procedural or immediate post‐procedural complications were observed.

## 3 Discussion

This case report demonstrates the successful application of the AR system for a percutaneous splenic mass biopsy performed in a minor procedure room. The procedure, which can be challenging due to the spleen's anatomical location, mobility, and vascularity, was executed with precision and efficiency.

The integration of AR technology provided distinct advantages over conventional 2D ultrasound guidance. The real‐time holographic superposition of pre‐procedural CT data with live ultrasound offered an enhanced spatial understanding of the target and critical adjacent structures as compared to traditional techniques. This “X‐ray vision” capability allowed the proceduralist to navigate the needle path with increased confidence and precision, potentially reducing the risk of complications associated with inadvertent organ or vessel puncture. Furthermore, this added confidence resulted in anecdotally faster procedural time and, therefore, reduced sedation time and room utilization. This aligns with findings from other studies exploring AR in percutaneous procedures, which suggest comparable accuracy and reduced cognitive load compared to traditional guidance methods [3, 16].

A key implication of this case is the demonstrated feasibility of operationalizing advanced AR navigational technology in a minor procedure room. Historically, such image‐guidance systems have been confined to highly equipped operating rooms or angiography suites due to infrastructure requirements coupled with perceived complexity. This successful application in a less specialized setting suggests broader applicability for AR systems, potentially democratizing access to precision guidance for a wider range of percutaneous interventions and treatment settings.

## 4 Conclusion

The AR system effectively guided a percutaneous splenic mass biopsy in a minor procedure room, demonstrating enhanced anatomical visualization, improved procedural ergonomics, and successful target acquisition without complications. This case highlights the system's potential to improve precision and efficiency in challenging interventions and suggests a broader utility of AR technology in diverse clinical environments beyond conventional specialized suites.

## Author Contributions

**Alexander S. Misono:** conceptualization, data curation, formal analysis, funding acquisition, investigation, methodology, project administration, resources, software, supervision, validation, visualization, writing – original draft, writing – review and editing.

## Funding

MediView provided funding for publication fees.

## Ethics Statement

As a single case report with the patient's signed consent, no other ethical review was required.

## Consent

Written informed consent was obtained from the patient for the publication of this case report.

## Conflicts of Interest

Author is a member of the Scientific Advisory Board for MediView XR Inc.

## Data Availability

Data sharing is not applicable to this article as no datasets were generated or analyzed for this case report. All relevant data have been shared in this manuscript.
